# Robust Torque Predictions From Electromyography Across Multiple Levels of Active Exoskeleton Assistance Despite Non-linear Reorganization of Locomotor Output

**DOI:** 10.3389/fnbot.2021.700823

**Published:** 2021-11-03

**Authors:** Jacob A. George, Andrew J. Gunnell, Dante Archangeli, Grace Hunt, Marshall Ishmael, K. Bo Foreman, Tommaso Lenzi

**Affiliations:** ^1^NeuroRobotics Lab, Department of Electrical and Computer Engineering, College of Engineering, University of Utah, Salt Lake City, UT, United States; ^2^NeuroRobotics Lab, Division of Physical Medicine and Rehabilitation, School of Medicine, University of Utah, Salt Lake City, UT, United States; ^3^Bionic Engineering Lab, Department of Mechanical Engineering, College of Engineering, University of Utah, Salt Lake City, UT, United States; ^4^Motion Analysis Facility, Department of Physical Therapy and Athletic Training, College of Health, University of Utah, Salt Lake City, UT, United States

**Keywords:** powered exoskeleton, hip orthosis, electromyography (EMG) control, adaptive control, wearable robotics, torque prediction, locomotor output

## Abstract

Robotic exoskeletons can assist humans with walking by providing supplemental torque in proportion to the user's joint torque. Electromyographic (EMG) control algorithms can estimate a user's joint torque directly using real-time EMG recordings from the muscles that generate the torque. However, EMG signals change as a result of supplemental torque from an exoskeleton, resulting in unreliable estimates of the user's joint torque during active exoskeleton assistance. Here, we present an EMG control framework for robotic exoskeletons that provides consistent joint torque predictions across varying levels of assistance. Experiments with three healthy human participants showed that using diverse training data (from different levels of assistance) enables robust torque predictions, and that a convolutional neural network (CNN), but not a Kalman filter (KF), can capture the non-linear transformations in EMG due to exoskeleton assistance. With diverse training, the CNN could reliably predict joint torque from EMG during zero, low, medium, and high levels of exoskeleton assistance [root mean squared error (RMSE) below 0.096 N-m/kg]. In contrast, without diverse training, RMSE of the CNN ranged from 0.106 to 0.144 N-m/kg. RMSE of the KF ranged from 0.137 to 0.182 N-m/kg without diverse training, and did not improve with diverse training. When participant time is limited, training data should emphasize the highest levels of assistance first and utilize at least 35 full gait cycles for the CNN. The results presented here constitute an important step toward adaptive and robust human augmentation via robotic exoskeletons. This work also highlights the non-linear reorganization of locomotor output when using assistive exoskeletons; significant reductions in EMG activity were observed for the soleus and gastrocnemius, and a significant increase in EMG activity was observed for the erector spinae. Control algorithms that can accommodate spatiotemporal changes in muscle activity have broad implications for exoskeleton-based assistance and rehabilitation following neuromuscular injury.

## Introduction

Robotic exoskeletons can assist humans with walking by providing supplemental torque at the joint level. Supplemental torque has been provided during hip flexion and/or extension to restore function after neuromuscular disability (Awad et al., 2017, 2020; Ishmael et al., 2019) or to increase metabolic efficiency for healthy individuals (Kim et al., 2019). In the case of healthy individuals, most assistive exoskeletons aim to provide assistive torque proportionate to the user's joint torque. Temporal alignment of the user's joint torque and the assistive torque provided by the exoskeleton is critical to the efficacy of the exoskeleton (Ding et al., 2018).

One approach to temporally align the user's joint torque and the assistive torque provided by the exoskeleton is to use electromyographic (EMG) control algorithms to estimate the user's joint torque directly using real-time EMG recordings from the muscles that generate the torque. Electromyographic activity precedes the resultant joint torque and kinematic motion, thereby allowing joint torque to be estimated before it is generated. Electromyographic control is traditionally established by collecting a dataset of synchronized EMG recordings and known joint torques, and then training an algorithm to predict torque from EMG under a supervised learning paradigm. During run-time operation, live EMG signals are used to predict joint torque in real-time.

However, a fundamental challenge with EMG-control strategies is that EMG activity changes as a result of active exoskeleton assistance. For example, active exoskeleton assistance that reduces metabolic cost is assumed to be reducing muscle effort (Ferris and Lewis, 2009; Kim et al., 2019), and a reduction in muscle effort is detectable with EMG recordings (Gordon et al., 2013). These changes in muscle effort are often non-linear and are observed in muscles which are not associated directly with the joint being assisted; for example, active exoskeleton assistance at the hip joint has been shown to reduce muscle effort at the ankle joint (Lenzi et al., 2013). In essence, the act of controlling the exoskeleton (i.e., assisting the user) changes the relationship of the control signal (EMG) to the desired torque. As a result, traditional EMG control algorithms provide unreliable estimates of the user's joint torque during active exoskeleton assistance.

Before EMG-controlled exoskeletons can be used in a real-world setting with a variety of locomotion modes, EMG control algorithms need to be robust to the non-linear changes in EMG that occur as a result of active exoskeleton assistance. Working toward that goal, here we sought to first identify an EMG-control framework that would provide reliable predictions of joint torque across a variety of different levels of active exoskeleton assistance during treadmill ambulation. We approached this challenge from a data-driven perspective in order to identify the data-collection practices and algorithms that result in the most accurate and robust torque predictions. Data were collected from three participants walking at consistent speeds with varying levels of exoskeleton assistance, and we assessed the error associated with torque predictions from linear and non-linear EMG-control algorithms across a variety of assistance levels.

Our results confirm non-linear reorganization of locomotor output due to active exoskeleton assistance and present a novel EMG-control framework for robotic exoskeletons that provides consistent joint torque estimates across varying levels of exoskeleton assistance. These results constitute an important step toward adaptive exoskeletons capable of assisting individuals reliably across varying levels of exoskeleton assistance.

## Materials and Methods

### Human Participants

Three healthy human participants—P1, P2, and P3—participated in this study. All participants were male, under 30 years old, and had prior training using a hip exoskeleton device. All experiments were carried out with informed consent from the participants and following protocols approved by the University of Utah Institutional Review Board.

### Hip Exoskeleton

**Figure 1:**
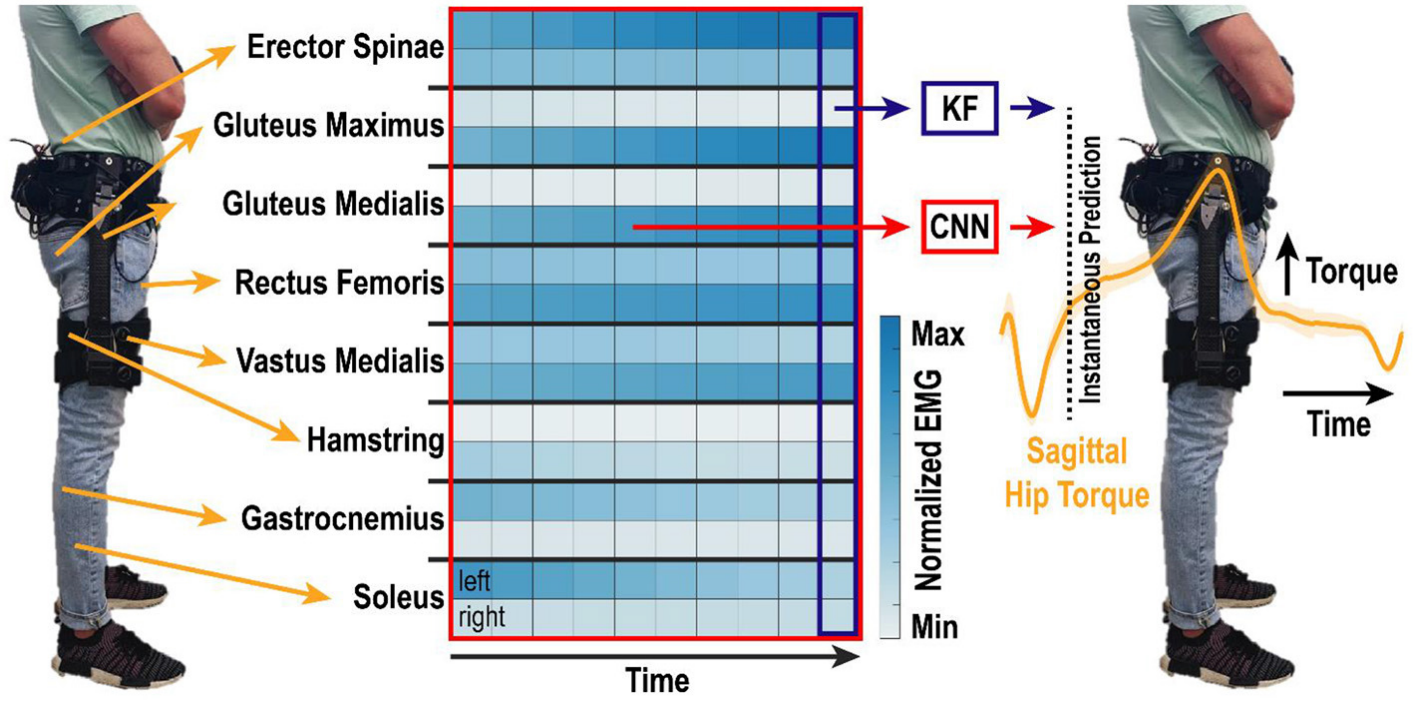
Algorithm overview. EMG was recorded bilaterally from eight lower-limb muscle groups and motion capture was used to determine lower-limb kinematics and kinetics. EMG activity from the one most recent or ten most recent samples in time were used by the KF and CNN, respectively, to predict instantaneous torque of the right and left hips in the sagittal direction. Samples were analyzed at 200 Hz, such that the total time window shown is 50 ms. Heatmap shows example spatiotemporal EMG activity during walking. Differences across muscle groups (rows of two), between left and right legs (top and bottom rows, respectively), and over time (columns) create a rich set of features that are non-linearly correlated to hip torque.

This study used a powered bilateral hip exoskeleton to assist walking (Figure 1). The exoskeleton design has been reported previously (Ishmael et al., 2019) and is summarized here briefly. Hip flexion and extension assistance in the sagittal plane were provided by an actuated joint. The actuator was connected to the participant through passive joints located distal and proximal to the powered joint. The passive joints were used to aid with initial alignment with the user's leg and allow voluntary frontal-plane motions during ambulation. The exoskeleton was attached to a flexible pelvis orthosis fabricated from polyurethane using 3D printing.

Custom control electronics were housed with a six-cell lithium-ion battery in a 3D-printed case on the lower back of the user. Sensing and motor power cables connected each actuator module of the bilateral exoskeleton to the control board. Onboard microcontrollers and an embedded computer performed the middle and high-level control computations to calculate a desired assistive joint torque (Ishmael et al., 2019). The desired torques were sent to onboard motor drivers, which performed low-level current control and output motor commutation signals to the actuation system. The assistive torque profile was generated from the summation of two Gaussian profiles—one for extension and another for flexion. An experimenter tuned the peak flexion and extension assistance timing by communicating with the custom control electronics over Wi-Fi.

### Experimental Conditions

Participants walked on a split-belt (each belt was 20-in wide) Bertec Fully Instrumented Treadmill (Bertec, Columbus, OH, USA) at 1.16 m/s. A fixed speed was used in order to force users to achieve similar kinematic profiles despite varying levels of exoskeleton assistance. A speed of 1.16 m/s was selected as approximately halfway between medium and slow walking speeds reported in Bovi et al. (2011). Participants walked for 1 min under each of the five following conditions: (1) *Baseline*—the participant walking normally without wearing the exoskeleton; (2) *No Assistance (Passive)*—the participant walking while wearing the exoskeleton in a powered state in which it provided no assistance but actively minimized resistance; (3) *Low Assistance*—the exoskeleton provided 6 N-m of assistive flexion and 2.5 N-m of assistive extension during walking; (4) *Medium Assistance*—the exoskeleton provided 9 N-m of assistive flexion and 3.75 N-m of assistive extension during walking; and (5) *High Assistance*—the exoskeleton provided 12 N-m of assistive flexion and 5 N-m of assistive extension during walking. The assistive torque was provided as absolute torque and was not normalized based on the individual participants mass or body-segment lengths. The order of the five conditions was randomized for each participant to minimize the effect of fatigue toward the latter trials due to extended periods of walking. In the days prior to the experiment, the participants practiced using the exoskeleton with low, medium, and high assistive torque.

### Motion Capture and Biomechanics

Each participant wore tight-fitting clothing with reflective markers representing a modified Plug-in-Gait Model with extra redundant markers placed on the pelvis for better tracking. Additional markers were placed on the hip exoskeleton: four on the exoskeleton's frame and four on the exoskeleton's passive degrees of freedom. The participant's pelvis and the hip exoskeleton's belt harness were assumed to move together as a rigid body. The hip exoskeleton's belt harness was kept on during all trial conditions (including the baseline, no-exoskeleton condition) in order for the reflective marker placement to remain the same throughout all trials. The participant's upper body was secured to a harness which was connected to an overhead support system.

Three calibration routines were performed with each participant. First, a static calibration was performed in which the participant was asked to stand still for 5 s with their feet shoulder width apart and arms bent out from their body. The static calibration was used to scale the Vicon Nexus and Visual 3D models for each subject. Second, a functional calibration was performed in which the participant walked at 1.16 m/s on the treadmill for approximately 5 s. The functional calibration was used to improve automatic marker labeling in Vicon Nexus. Third, a joint center calibration was performed in which the participant was asked to swing their legs in a clock pattern and perform two squats to bend their knees. The joint center calibration was used to locate the subject's knee joint centers using Symmetrical Axis of Rotation Analysis (SARA). Following the calibrations, each participant walked under the five experimental conditions described above. Marker trajectories and ground reaction force data were synchronized, recorded, and pre-processed using Vicon Nexus 2 software. The marker trajectory data was collected at 200 Hz and the ground reaction force data at 1,000 Hz. Heel-strike gait events were identified when each ground reaction force exceeded a threshold of 30 N. After pre-processing was completed, the trajectories, analog data from the force plates and EMG signals, and gait events were imported into Visual 3D software. A low-pass Butterworth filter with a cut-off frequency of 6 Hz was applied to the marker trajectories and another low-pass Butterworth filter with a cut-off frequency of 15 Hz was applied for the analog force plate data. Because of inconsistencies between each subject's hip joint center using Symmetrical Center of Rotation Estimation (SCoRE), landmarks were created for each subject's hip joint center of rotations using distances between anterior superior iliac spine pelvis markers and iliac crest markers. Using the joint center of rotations, we computed the kinetics and kinematics of the ankle, knee, and hip for both legs. The kinetics and kinematics were calculated in Visual 3D and then exported as MATLAB files for each participant.

### EMG Acquisition

Surface EMG was recorded from 16 channels using the 16-channel MA400 EMG Motion Lab System. The following muscle groups were targeted based on the locations recommended by the SENIAM project (SENIAM, 2021): soleus, gastrocnemius, hamstring, gluteus maximus, gluteus medius, vastus medialis, rectus femoris, and erector spinae. The skin was shaved and cleaned with rubbing alcohol prior to placing the surface electrodes. Electrodes were held in place using self-adherent wrap (Coban, 3M; Saint Paul, MN, USA). Electromyographic signals were sampled at 3,000 Hz and band-pass filtered with cutoff frequencies of 20 and 450 Hz in MATLAB. The rectified EMG signal was then low-pass filtered at 15 Hz in MATLAB.

### Data Analysis

Kinetics, kinematics, and EMG data were synchronized for each participant at 200 Hz. Data were segmented into strides from heel strike to heel strike (defined as when the ground reaction force exceeded 30 N). The kinetics, kinematics, and EMG for each gait event were resampled to 1,000 samples for visual overlays. Algorithm training data consisted of continuous EMG data from 16 channels and the continuous combined hip torque of the human-exoskeleton system in the sagittal direction for the right and left legs.

### EMG Analysis

For each electrode, the mean and peak EMG were calculated for each individual gait cycle for both the left and right legs. The mean and standard error of the mean were calculated for the mean and peak EMG across all of the gait cycles for each of the five experimental conditions. Electromyographic activity was then normalized relative to the baseline condition. This process was completed independently for each participant.

### Algorithms and Training Conditions

Two algorithms were implemented in MATLAB to estimate hip torque from the 16 continuous EMG features: a standard Kalman filter (KF) (George et al., 2019) and a convolutional neural network (CNN) (George et al., 2018) (Figure 1). Both algorithms have been used extensively before for real-time myoelectric control of bionic devices (George et al., 2018, 2019, 2020; Brinton et al., 2020; Paskett et al., 2021), and are summarized here briefly.

The KF provides an efficient recursive algorithm to optimally estimate the posterior probability of hip torque when the likelihood model (i.e., the probability of the EMG activity given current hip torque) and prior models (i.e., the state model of how torques change over time) are linear and Gaussian. The inclusion of prior information about the system state enables an efficient recursive formulation of the decoding algorithm and effectively smooths noisy estimates in a mathematically principled way (Wu et al., 2006). In the implementation presented here, the KF predicts the instantaneous torque of the right and left hip in the sagittal plane based on the EMG activity at the current time-point (Figure 1).

**Figure 2:**
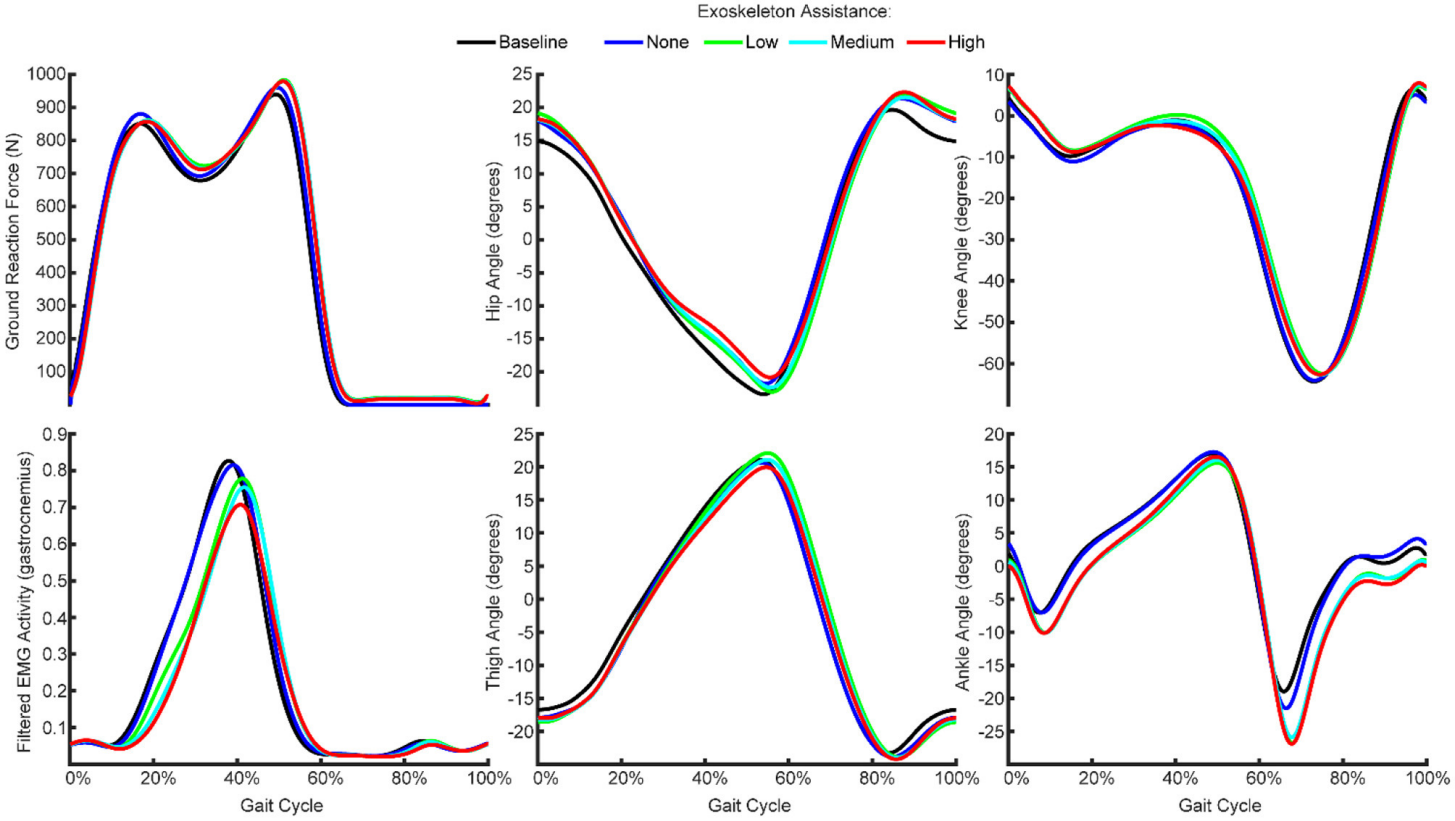
Mean ground reaction force, kinematics and EMG features across all gait cycles from one participant recorded during the baseline condition and with exoskeleton assistance set to none, low, medium, or high. The participants walked at a fixed speed with varying levels of exoskeleton assistance, such that kinematics and gait were generally consistent while EMG activity generally decreased with increasing exoskeleton assistance.

In contrast, the CNN predicts the instantaneous torque of the right and left hip in the sagittal plane based on a spatiotemporal “image” of EMG activity over the last 10 samples in time (George et al., 2018) (Figure 1). The CNN utilizes convolution to learn complex spatiotemporal relations within EMG activity that correlate to torque output. A series of non-linear weights are used to map the spatiotemporal features extracted from the convolution to the instantaneous torque. The architecture of the CNN is defined in (George et al., 2018) and the specific instantiation for this manuscript was as follows: The CNN input was an 16 × 10 image consisting of 16 EMG features sampled at the current time and nine previous time points. The CNN architecture (Figure 2) consisted of a single convolutional layer, two fully-connected layers, ReLu activation between layers and a regression output. A 1 × 5 kernel was used for convolution, such that the convolution was only across time and not across the feature set. A total of 10 convolutional filters were used to produce a 16 × 6 × 10 output feature map. The output of the convolutional layer was then passed through a ReLu activation layer before being passed to the first fully-connected layer. The output of the first fully-connected layer was also passed through a ReLu activation layer before being passed to the second fully-connected layer. Both fully-connected layers consisted of 1,056 neurons. The output of the second fully-connected layer was then fed into a final fully-connected layer that produced a total of two regression outputs, for the right and left hip torque in the sagittal plane. The CNN was trained using a Stochastic Gradient Descent with Momentum solver with an initial learning rate of 0.1 and a piecewise learning rate drop factor of 50% every 10 epochs. Training continued until 20 epochs had passed with no increase in performance on the validation data, or after a maximum of 2,000 epochs had passed.

A total of six KFs and six CNNs were trained under six different training conditions: (1–5) using independent data from each of the five experimental conditions, and (6) using combined data from all five of the experimental conditions. The KFs were trained using 75% of the gait cycles for given condition. The CNNs were trained using 65% of the gait cycles and validated using 10% of the gait cycles for a given condition. Each algorithm was tested on the remaining 25% of the gait cycles (not used for training) for each of the six conditions. Performance was measured by the root mean squared error (RMSE) between the algorithm prediction and the true torque of the participant.

To determine the impact of the number of gait cycles trained on, performance was assessed for each algorithm, under each of the five experimental conditions, by iteratively increasing the number of gait cycles from two to the maximum number of gait cycles available from the training data. Additional gait cycles were added in the temporal sequence in which they occurred in the training data. Similarly, to determine the impact of the number of EMG channels using in the training data, performance was assessed for each algorithm, under each experimental condition, by iteratively increasing the number of EMG channels from 1 to 16. Additional EMG channels were added using a stepwise Gram-Schmidt channel-selection algorithm (Nieveen et al., 2017).

### Statistical Analyses

A separate one-way ANOVA was performed for each participant and each algorithm to compare the performance of the different training conditions. If any significance was found, subsequent pairwise comparisons (*t*-tests) were made using the Dunn-Sidák correction for multiple comparisons.

## Results

### Exoskeleton Assistance Caused Non-linear, Participant-specific Changes in EMG With Minimal Changes in Kinematics

We recorded kinematics, kinetics, and EMG activity while the participants walked normally, while wearing a passive exoskeleton, and while wearing the exoskeleton with low, medium, and high assistance (Figure 1). We found that the kinematics were generally consistent across the conditions (Figure 2). That is, the thigh, hip, knee, and ankle angles throughout the gait cycle did not change meaningfully as a result of wearing the exoskeleton or increasing the exoskeleton assistance.

**Figure 3:**
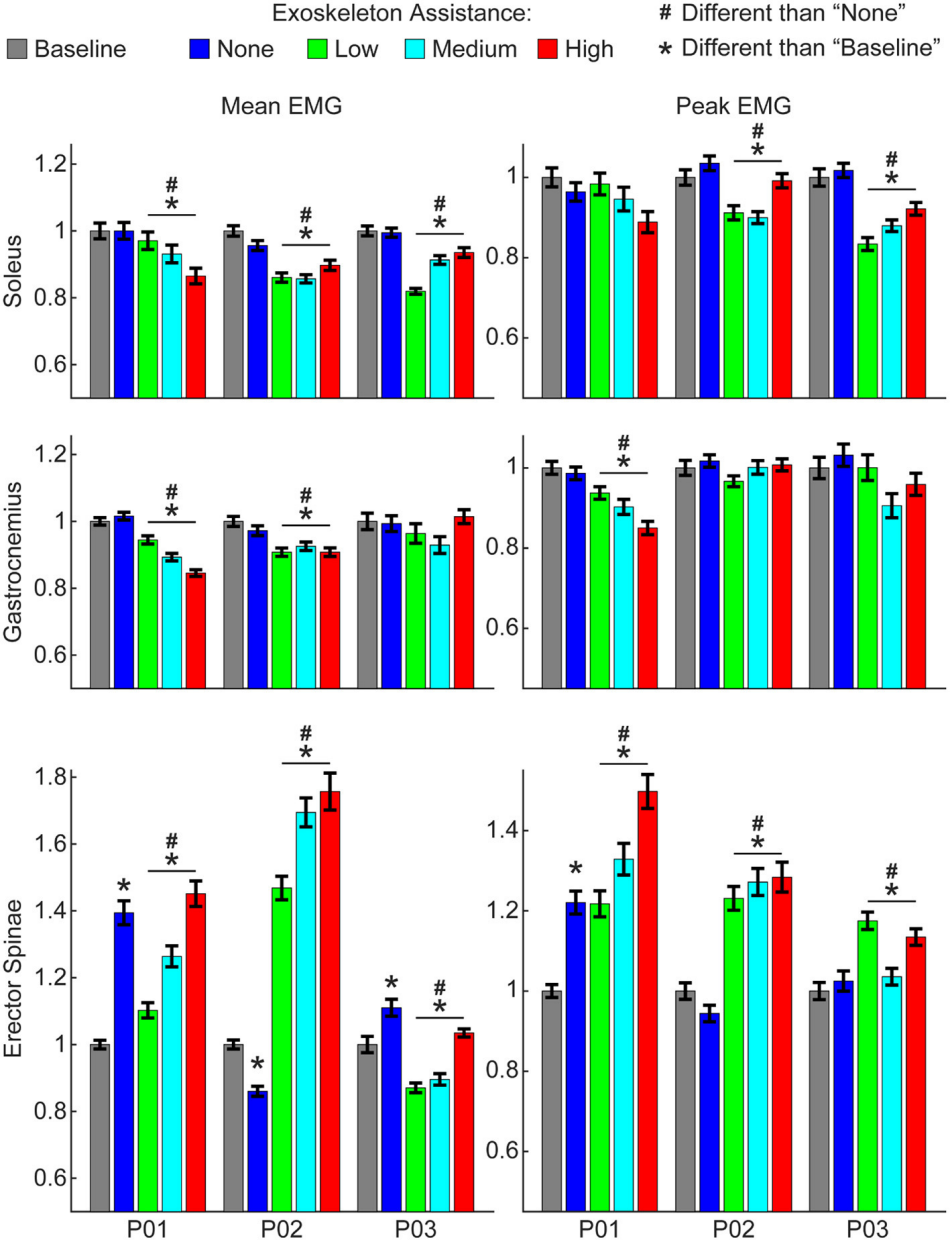
Changes in EMG activity due to exoskeleton assistance. Changes in EMG activity were non-linear and unique to each muscle group and participant. Generally, wearing the exoskeleton in a passive state “none” condition did not alter EMG activity of lower-limb muscles, but did increase activity of the lower-back (erector spinae) muscles. Mean and peak EMG activity for lower-limb muscles were significantly less with active exoskeleton assistance relative to the baseline and relative to the passive exoskeleton. In contrast, mean and peak EMG activity for lower-back muscles were significantly greater with active exoskeleton assistance relative to the baseline and relative to the passive exoskeleton. Trends with increasing exoskeleton assistance were unique to each participant and muscle group. Additional muscle groups are shown in Supplementary Figure 1. Data show EMG activity averaged across each gait cycle for the right and left legs and normalized to the baseline condition.

However, increasing exoskeleton assistance did lead to substantial non-linear changes in EMG activity. Most notably, active exoskeleton assistance resulted in a statistically significant decrease in mean soleus activity relative to the passive exoskeleton and baseline (normal walking) conditions for all three participants (Figure 3). Similarly, active exoskeleton assistance resulted in a statistically significant decrease in mean gastrocnemius activity relative to the passive exoskeleton and baseline (normal walking) conditions for two of the three participants. In contrast, active exoskeleton assistance resulted in a statistically significant increase in mean erector spinae activity relative to the passive exoskeleton and baseline (normal walking) conditions for all three participants. Changes in other muscles activity were less noticeable, and highly participant-specific (Supplementary Figure 1).

Increasing exoskeleton assistance from low to high did not systematically change EMG activity. Some trends were observed, but they were generally participant-specific and not statistically significant. For example, for P01 increasing exoskeleton assistance from low to high trended toward decreasing mean soleus activity, but the opposite trend was observed for P03, and virtually no difference was seen for P02.

### Algorithm Performance Does Not Extrapolate to Conditions Not Explicitly Trained On

**Figure 4:**
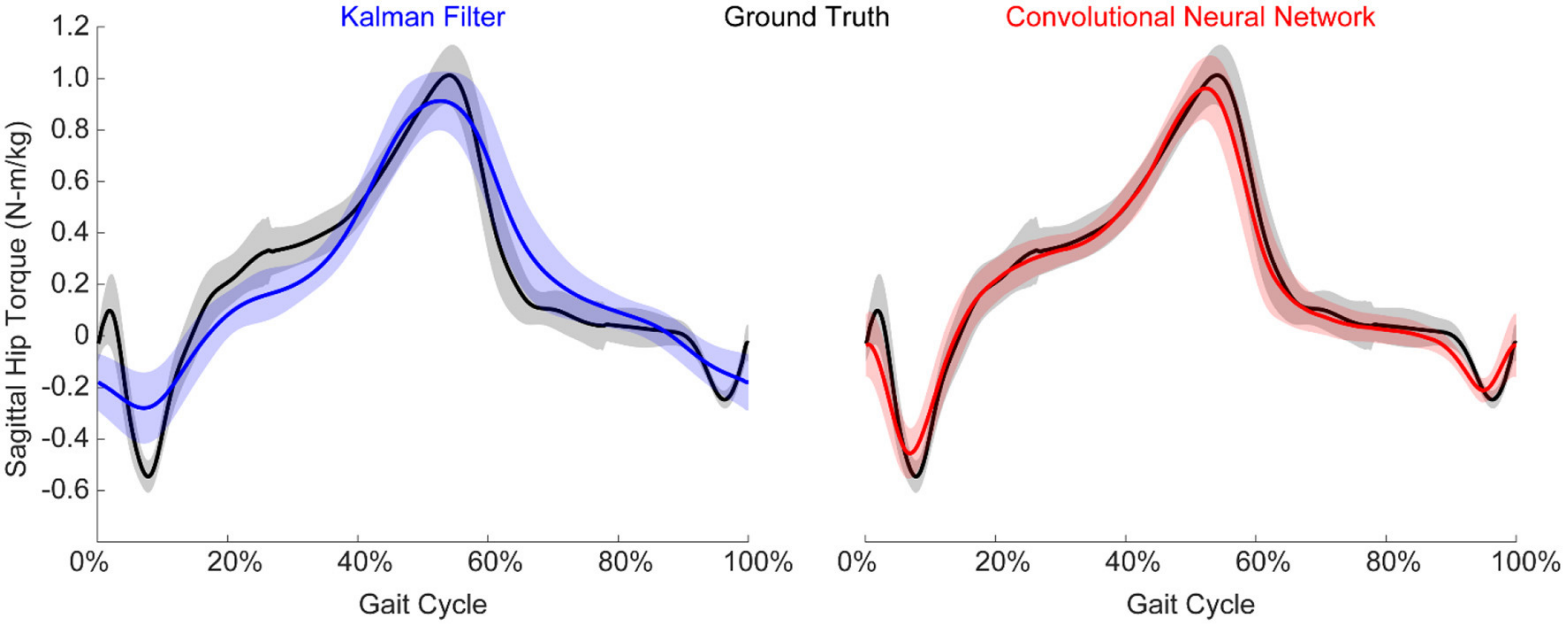
Example normalized torque predictions (N-m/kg) of the hip joint in the sagittal plane. Both algorithms were able to reliably estimate torque, although the CNN generally outperformed the KF. Figure shows mean ± standard deviation.

**Figure 5:**
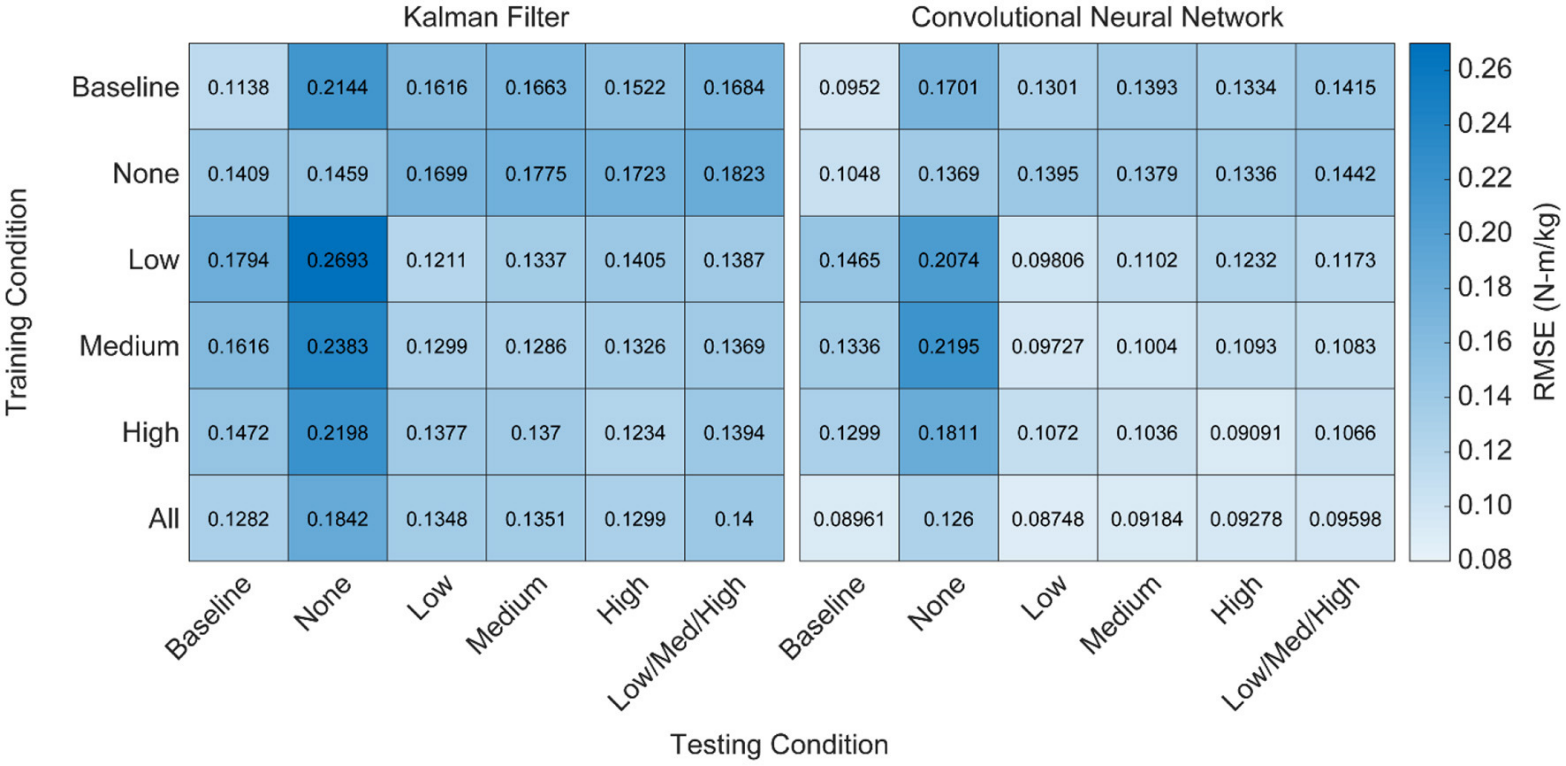
Heatmap of algorithm performance under various training and testing conditions. Performance is shown as RMSE (N-m/kg) of algorithm predictions relative to the ground truth values of right and left hip torque in the sagittal plane. Lighter colors represent lower RMSE and better performance. Training and testing on the same condition led to strong performance for both algorithms, as indicated by low RMSE values along the diagonals. Testing on conditions that were not explicitly trained on resulted in worse performance, as indicated by high RMSE values off the diagonals. Training on all of the conditions improved the overall performance of the CNN when testing on various levels of exoskeleton assistance, but did not improve the overall performance of the KF. That is, diverse training data increased the adaptability of the CNN, but not the KF.

We implemented a KF and a CNN to predict hip torque based on EMG activity. We explored the impact of training data on run-time performance of the algorithm by testing all possible training conditions on all possible testing conditions. The participant hip torque (ground truth) was generally consistent within a given condition, and both algorithms were able to accurately recreate the torque when trained using data from that same condition (Figure 4). However, when extrapolating to levels of exoskeleton assistance that were not explicitly trained on, the performance of both algorithms worsened (Figure 5). Predicting hip torque while the participant was walking with the exoskeleton in the passive configuration was generally the hardest condition to learn. The overall worst performance occurred when training on low exoskeleton assistance and then predicting hip torque while the participant was walking with the exoskeleton in the passive configuration. Training on the baseline condition or passive exoskeleton condition also generally resulted in poor performance when predicting hip torque while the participant was walking with active exoskeleton assistance.

### Training Data Should Be Collected During Active Exoskeleton Assistance

**Figure 6:**
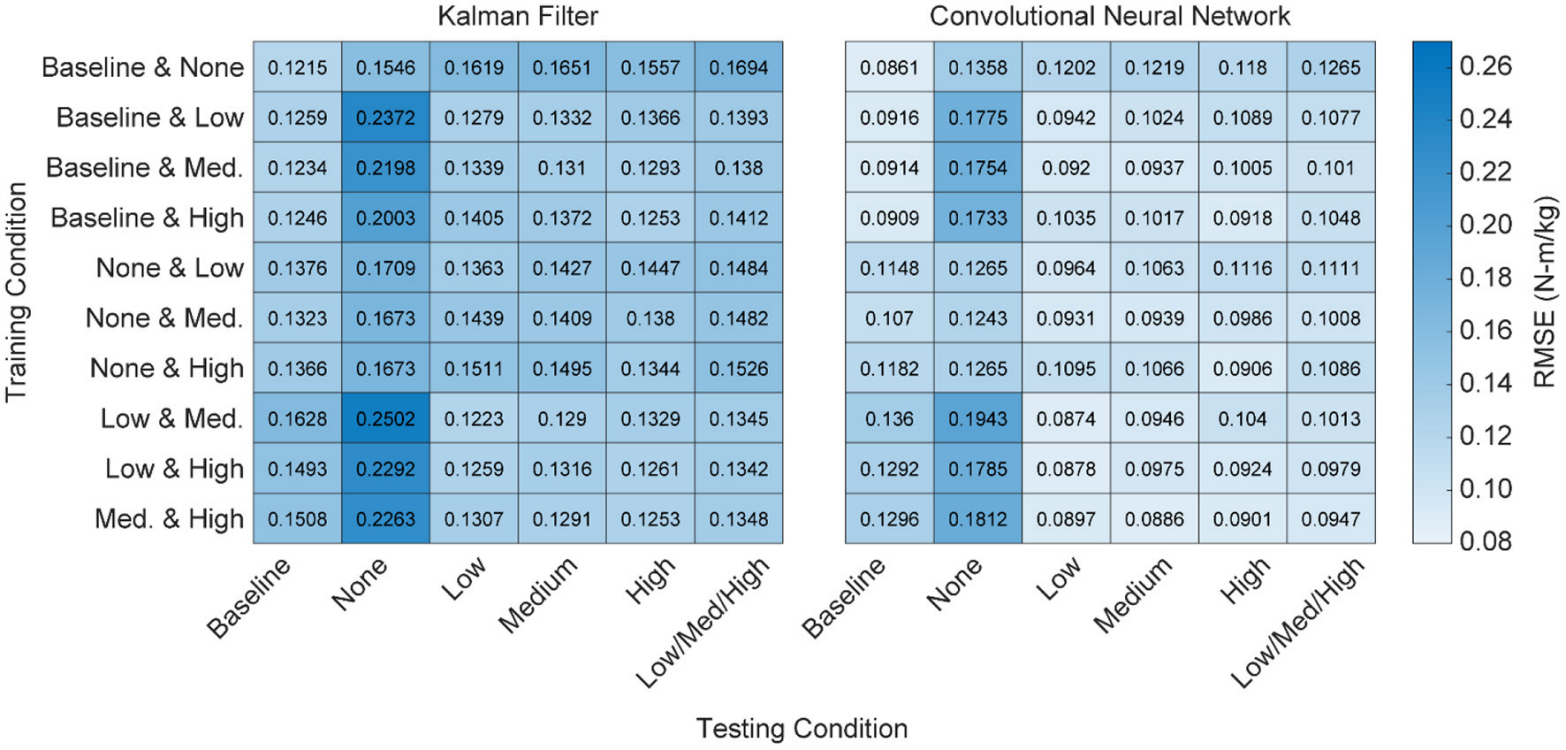
Heatmap of algorithm performance using various pairwise training approaches. Performance is shown as RMSE (N-m/kg) of algorithm predictions relative to the ground truth values of right and left hip torque in the sagittal plane. Lighter colors represent lower RMSE and better performance. Training with higher levels of exoskeleton assistance generally resulted in better performance. That is, when participant time is limited and only a subset of training conditions can be used, emphasis should be placed on higher levels of exoskeleton assistance.

From a practical perspective, torque only needs to be predicted during active exoskeleton assistance—that is, when the exoskeleton is providing a low, medium, or high level of assistance. To this end, we assessed the overall performance of the training conditions by looking at the combined RMSE across the low, medium and high levels of assistance (Figure 6). For both algorithms, training on the baseline and passive conditions resulted in the worst practical performance (*p*'s < 0.05). For the KF, there was no statistical differences when training on the low, medium, or high conditions. For the CNN, training on the medium and high conditions outperformed training on the low condition (*p*'s < 0.05).

### CNNs Trained on Diverse Training Data Perform Well Across All Levels of Assistance

Having demonstrated that algorithm performance does not extrapolate well to untrained levels of exoskeleton assistance, we assessed the performance of the algorithms when training data across all conditions is available—that is, the algorithm was trained using data from the baseline, passive/none, low, medium, and high conditions. We found that training on all of the conditions resulted in a statistically significant improvement for the CNN (*p*'s < 0.05), but not for the KF. Furthermore, training on all of the conditions resulted in the best overall practical performance for the CNN. Interestingly, adding training data not explicitly relevant to the subtask did not degrade the performance of the CNN (Figure 5). For example, training on all of the conditions and testing only on the low conditions was not worse than just training on the low condition alone. Thus, training a CNN on diverse training data can yield a multipurpose algorithm capable of performing well at a variety of assistance levels.

### When Participant Time Is Limited, Training Should Emphasis High Levels of Active Assistance

**Figure 7:**
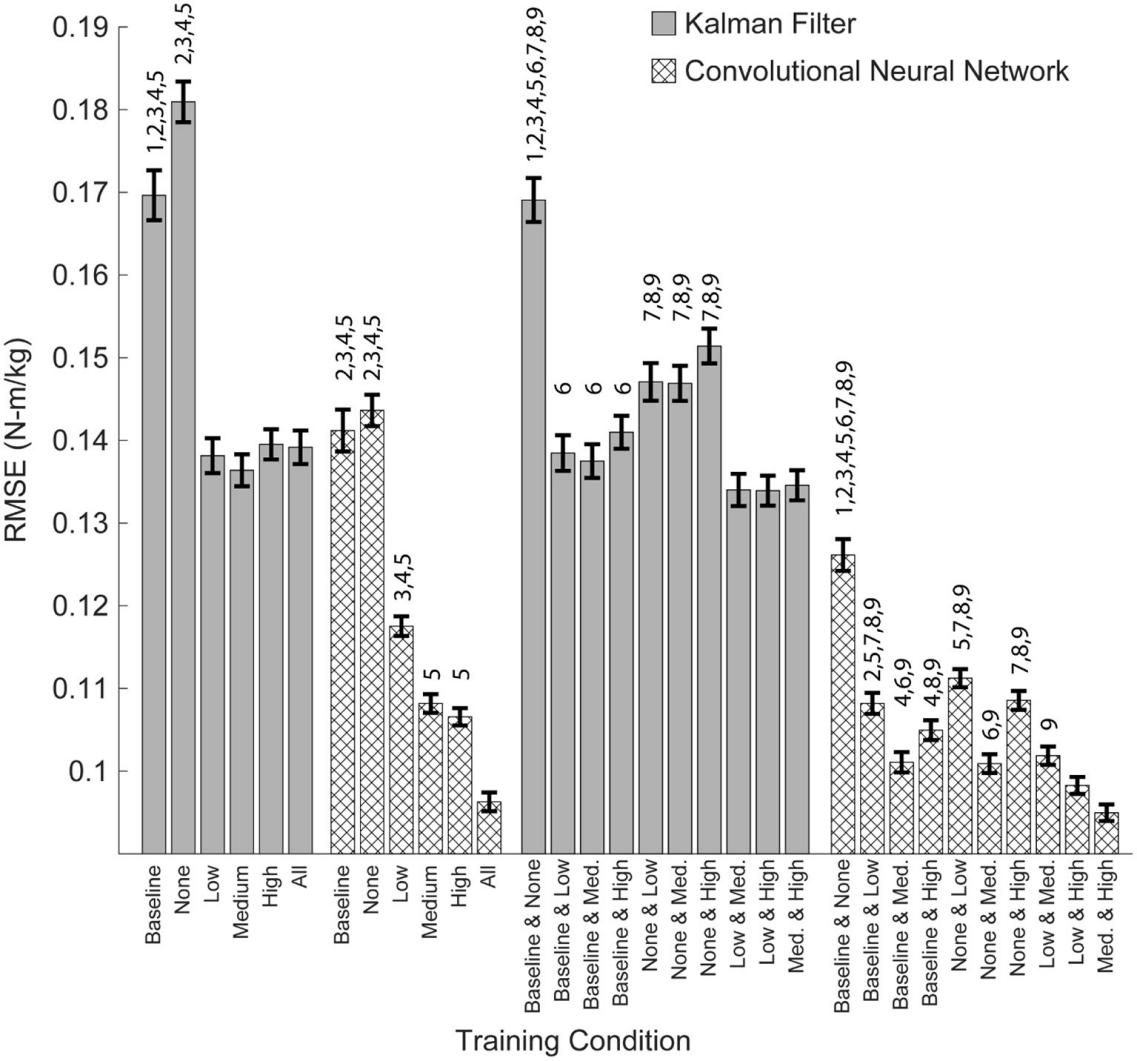
Overall algorithm performance when predicting torque during low, medium and high levels of exoskeleton assistance. Performance is shown as RMSE (N-m/kg) of algorithm predictions relative to the ground truth values of right and left hip torque in the sagittal plane. Training on data from multiple levels of exoskeleton assistance improved the overall performance of the CNN, but not the KF. Bars show mean ± standard error of the mean. Numbers above the bars denote statistical significance for the algorithm within the subset of adjacent conditions (multiple pair-wise comparisons using the Dunn-Sidak correction for multiple comparison). Conditions within the subsets are numbered from 0 to 6, left to right (Baseline = 0, High = 5), or from 0 to 9, left to right (Baseline and None = 0, Med. and High = 9).

Training on all possible conditions is ideal, but may not be practical when participant time is limited. To address this question, we looked at the performance of the algorithms when training on just two conditions and then testing on all possible conditions (Figure 7). As noted earlier, we found that training on the baseline and passive conditions resulted in the worst practical performance for both algorithms (Figure 6; *p*'s < 0.05). Similarly, for both the CNN and KF, the best performance was seen when training focused on active assistance (i.e., the low + medium, medium + high, or low + high conditions). For the CNN, training on the medium + high condition resulted in the best overall performance (*p*'s < 0.05 compared to all other pairwise conditions except the low + high condition).

### When Gait Cycles Are Very Limited, the KF Outperforms the CNN

**Figure 8:**
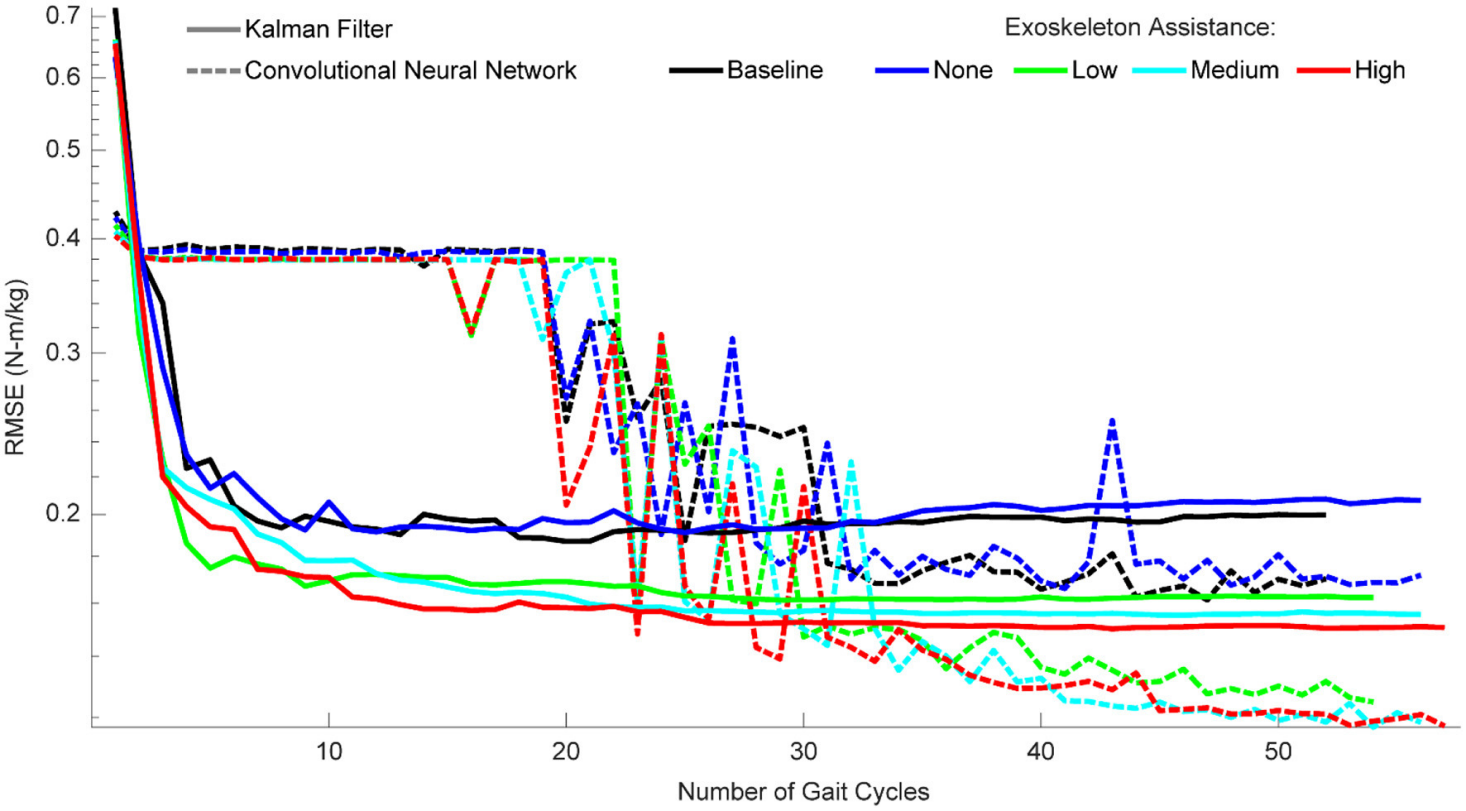
Algorithm performance as a function of the number of gait cycles trained on. Performance is shown as RMSE (N-m/kg) of algorithm predictions relative to the ground truth values of right and left hip torque in the sagittal plane. For the KF, training data should consist of a minimum of three gait cycles, and little improvement is seen after ten gait cycles. For the CNN, at least 20 gait cycles are necessary for the algorithm to begin improving, and at least 35 gait cycles are needed to reliably outperform the KF. CNN performance continues to improve with additional gait cycles, but only if those gait cycles involve some level of exoskeleton assistance.

When participant time is limited and diverse training data is encouraged, the amount of data needed for each condition becomes an important factor. To provide guidance on this, we quantified the performance of the algorithms as a function of the number of gait cycles trained on for each condition (Figure 8). For the KF, performance improved greatly within the first three gait cycles, and then leveled off after ~10 cycles. For the CNN, performance only began improving after 20 cycles and became much more reliable after ~35 cycles. After 35 cycles, performance leveled off for the baseline and passive conditions, but continued to improve for the active assistance conditions (up to the maximum number of gait cycles). In summary, with very limited data, we recommend a KF with at least three gait cycles, but ideally 10. When at least 35 gait cycles are available, we recommend a CNN—and encourage as much data as possible.

### Additional EMG Channels, Including Bilateral Pairs, Improve Accuracy

**Figure 9:**
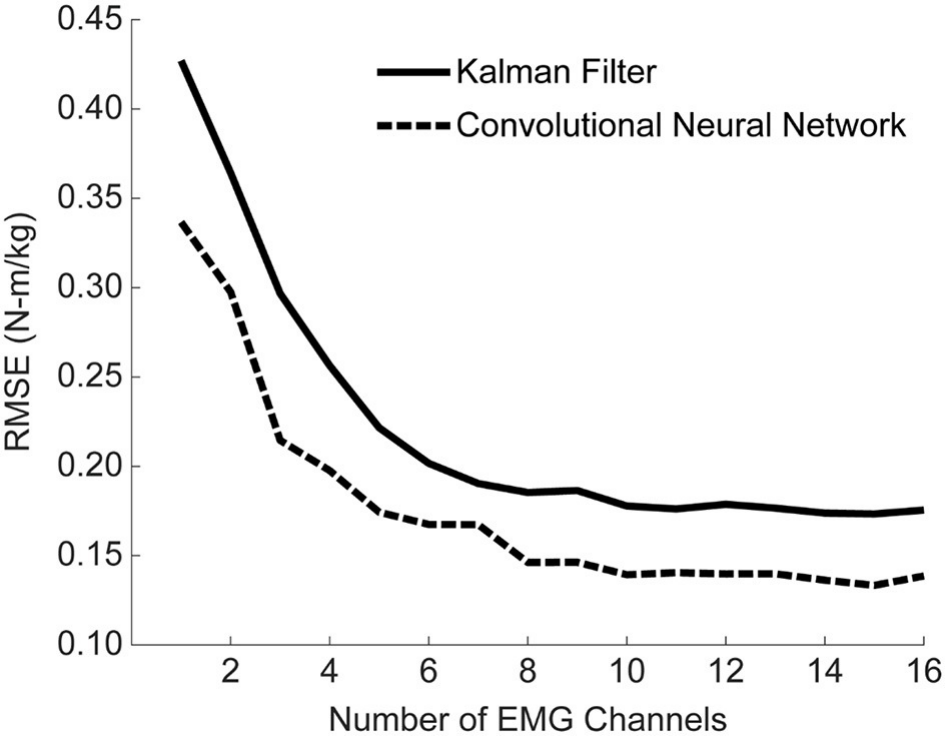
Mean algorithm performance across all training conditions as a function of the number of EMG channels used. Performance is shown as RMSE (N-m/kg) of algorithm predictions relative to the ground truth values of right and left hip torque in the sagittal plane. Channels were selected using a Gram-Schmitt channel selection algorithm. Both the KF and CNN improve with additional channels, although the majority of the improvement comes with the first eight channels. Note that the 16 channels used here consist of eight muscles groups recorded bilaterally, although the eight channels selected were rarely unilateral.

**Figure 10:**
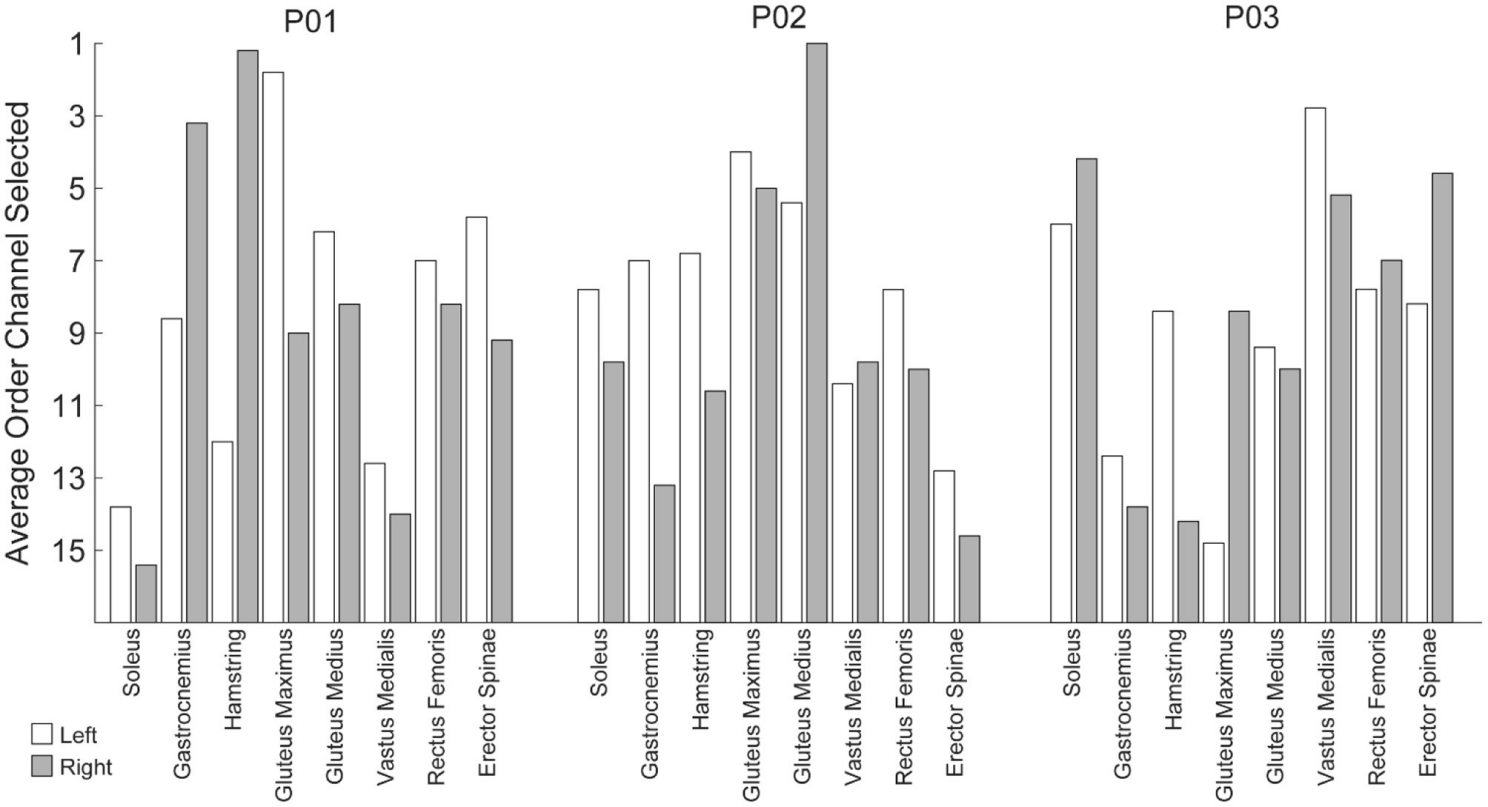
Average order in which EMG channels were selected when using a step-wise Gram-Schmitt channel-selection algorithm. The order channels were selected was unique per participant and generally resulted in bilateral pairs from a subset of muscle groups as opposed to maximizing unilateral independent muscle groups.

The analyses thus far utilized eight bilateral pairs of EMG. However, working toward practical implementation, we sought to measure the impact of the number of EMG channels on algorithm performance. To this end, we used a stepwise Gram-Schmidt channel-selection algorithm (Nieveen et al., 2017) to assess algorithm performance when sequentially adding the next best channel one by one. We found that both the algorithms improved with additional channels, although the majority of the improvement comes with the first eight channels (Figure 9). Despite the fact that there were eight bilateral pairs, the first eight channels selected were rarely unilateral (Figure 10). Selection order of EMG channels was also highly participant-specific. For example, the right and left soleus muscles were consistently selected last for P01, while the right and left soleus were among the first five selected for P03.

## Discussion

This work serves as one of the first demonstrations of adaptive EMG control across varying levels of exoskeleton assistance. Consistent with prior results, we show a non-linear reorganization of locomotor output due to active exoskeleton assistance (Gordon et al., 2013; Lenzi et al., 2013; Sylos-Labini et al., 2014). We also show that, with appropriate training data, a CNN can capture these non-linear changes and accurately predict torque while a KF cannot. This finding is particularly relevant given the extensive use of KFs for predicting joint torque (Menegaldo, 2017; Teramae et al., 2018; Lyu et al., 2019).

Prior work has shown that neural network algorithms trained on mechanical sensor data are capable of generalizing predictions of hip torque across cyclic ambulation modes that were not explicitly trained on when using a fixed level of exoskeleton assistance (Molinaro et al., 2020). Here, we demonstrate that neural network algorithms trained on EMG recordings are *not* capable of generalizing predictions of hip torque across varying levels of exoskeleton assistance without explicit training on those levels of assistance. We saw a significant improvement in algorithm performance when training on additional levels of assistance, while (Molinaro et al., 2020) saw no significance difference when training on additional cyclic ambulation modes. Taken together, this suggests that data-driven machine-learning approaches to control lower-limb exoskeletons should emphasize training on various levels of assistance, but may not necessarily need to train on all possible cyclic ambulation modes (e.g., ascending/descending stairs, walking on level ground). We speculate the ability of control algorithms to generalize across cyclic ambulation modes is due to the fact that changes in ambulation mode result in relatively linear changes in locomotor output (e.g., a shift in the amplitude, density, or offset of the torque profile). In contrast, as demonstrated here, there is a highly non-linear reorganization of EMG activity that occurs with different levels of exoskeleton assistance. Future work should investigate whether or not EMG control algorithms can generalize to acyclic ambulation when trained on cyclic ambulation.

Due to the limited number of participants utilized in this study and prior studies (Molinaro et al., 2020), it is still unclear if neural network models can generalize across participants. That said, the results presented here provide a realistic path forward to participant-specific models of EMG control. When participant time is limited, training should emphasize training on the highest levels of assistance with at least eight EMG channels and at least 35 gait cycles. If <35 gait cycles are available, a KF should be utilized over a CNN. Although we did not use the algorithms presented here for real-time human-in-the-loop control, prior work has demonstrated that the KF and CNN used in this study are fast enough to be run in real-time with minimal computational resources and still provide stable control during complex activities of daily living (George et al., 2018, 2020; Brinton et al., 2020; Paskett et al., 2021). Future work should implement these algorithms for real-time human-in-the-loop EMG control of the hip exoskeleton presented here and address how improvements in torque prediction accuracy translate to real-time control and user comfort.

The error reported with the sagittal hip torque estimates presented here are favorable relative to prior work. Prior RMSEs of sagittal hip torque during level ground walking have been reported as 0.15 N-m/kg using pressure insoles (Forner-Cordero et al., 2006), 0.093 N-m/kg using mechanical sensors (Molinaro et al., 2020), and 0.20 N-m/kg using demographic, anthropometric, kinematic, and EMG data (Hahn and O'Keefe, 2011). In comparison, the best RMSE reported here was 0.095 N-m/kg using strictly EMG data.

Using only EMG data for exoskeleton control offers the unique ability to predict joint torque before any physical movements occur. Traditional approaches, like those mentioned above, have relied on onboard mechanical sensors (e.g., force sensors, inertial measurement units) to detect changes in gait cadence, slope, or task. Use of mechanical sensors is limited in that mechanical sensors can only detect a user's motor intent after their action has occurred, thereby making them prone to considerable mechanical delays (Ferris et al., 2007). In contrast, EMG precedes physical movement and therefore offers the future possibility of detecting novel actions before they happen (e.g., changing direction or starting/stopping walking).

Prior work has shown that users can adapt to simple linear proportional models for EMG control to maintain movement accuracy (Lenzi et al., 2012) and reduce overall energy expenditure (Gordon et al., 2013; Ao et al., 2017). The work presented here builds on these foundational studies by introducing robust data-driven machine-learning approaches that allow EMG control to provide more accurate estimates of torques from non-linear muscle groups. For example, simple linear proportional models work best when there is a primary muscle that is solely responsible for torque generation, like the bicep for the elbow (Lenzi et al., 2012) or the soleus for the ankle (Gordon et al., 2013; Ao et al., 2017). More advanced controllers are required to predict complex torque profiles (e.g., hip torque) from non-linear muscle activity.

Altogether, this work provides practical contributions toward robust EMG control of exoskeletons and has broad implications for the field of rehabilitation robotics. Electromyographic control algorithms that are robust to changes in EMG can ultimately be used to assist individuals when EMG changes as a result of neuromuscular impairment, or to guide rehabilitation when EMG changes as a result of neuromuscular recovery.

## Data Availability Statement

The original contributions presented in the study are included in the article/supplementary material, further inquiries can be directed to the corresponding author/s.

## Ethics Statement

The studies involving human participants were reviewed and approved by University of Utah Institutional Review Board. The patients/participants provided their written informed consent to participate in this study. Written informed consent was obtained from the individual(s) for the publication of any potentially identifiable images or data included in this article.

## Author Contributions

JG designed experiments, collected data, developed machine-learning algorithms, analyzed data, and wrote the manuscript. AG and GH collected data, developed biomechanical models, and processed motion-capture data. DA and MI collected data, designed, developed, and controlled the hip exoskeleton. KF oversaw data collection and motion capture analyses. TL oversaw all aspects of the work. All authors contributed to the revision of the manuscript.

## Funding

Research reported in this publication was supported by the Department of Defense (DOD), Congressionally Directed Medical Research Programs (CDMRP), Peer Reviewed Orthopedic Research Program (PRORP), Applied Research Award (ARA), contract number W81XWH-16-1-0701 awarded to TL. Research reported in this publication was also supported by the Office of The Director (OD), Eunice Kennedy Shriver National Institute of Child Health and Human Development (NICHD), and National Institute of Dental and Craniofacial Research (NIDCR) of the National Institutes of Health (NIH) under Award Number DP5OD029571 awarded to JG. Additional trainee support for JG was provided by the University of Utah Center for Clinical and Translational Science (CCTS) Interdisciplinary Spheres of Translation Across the Research Spectrum (STARS) TL1 Training Program, with funding from the National Center for Advancing Translational Sciences of the National Institutes of Health under Award Number UL1TR002538 and TL1TR002540.

## Author Disclaimer

The content is solely the responsibility of the authors and does not necessarily represent the official views of the National Institutes of Health or the Department of Defense.

## Conflict of Interest

The authors declare that the research was conducted in the absence of any commercial or financial relationships that could be construed as a potential conflict of interest.

## Publisher's Note

All claims expressed in this article are solely those of the authors and do not necessarily represent those of their affiliated organizations, or those of the publisher, the editors and the reviewers. Any product that may be evaluated in this article, or claim that may be made by its manufacturer, is not guaranteed or endorsed by the publisher.
