# Supplementary material for: Robust Torque Predictions From Electromyography Across Multiple Levels of Active Exoskeleton Assistance Despite Non-linear Reorganization of Locomotor Output
Source: Front Neurorobot. 2021 Nov 3;15:700823. doi: 10.3389/fnbot.2021.700823 (PMC8595105; doi:10.3389/fnbot.2021.700823)
Supplement: Supplementary file 1 [file Data_Sheet_1.docx]

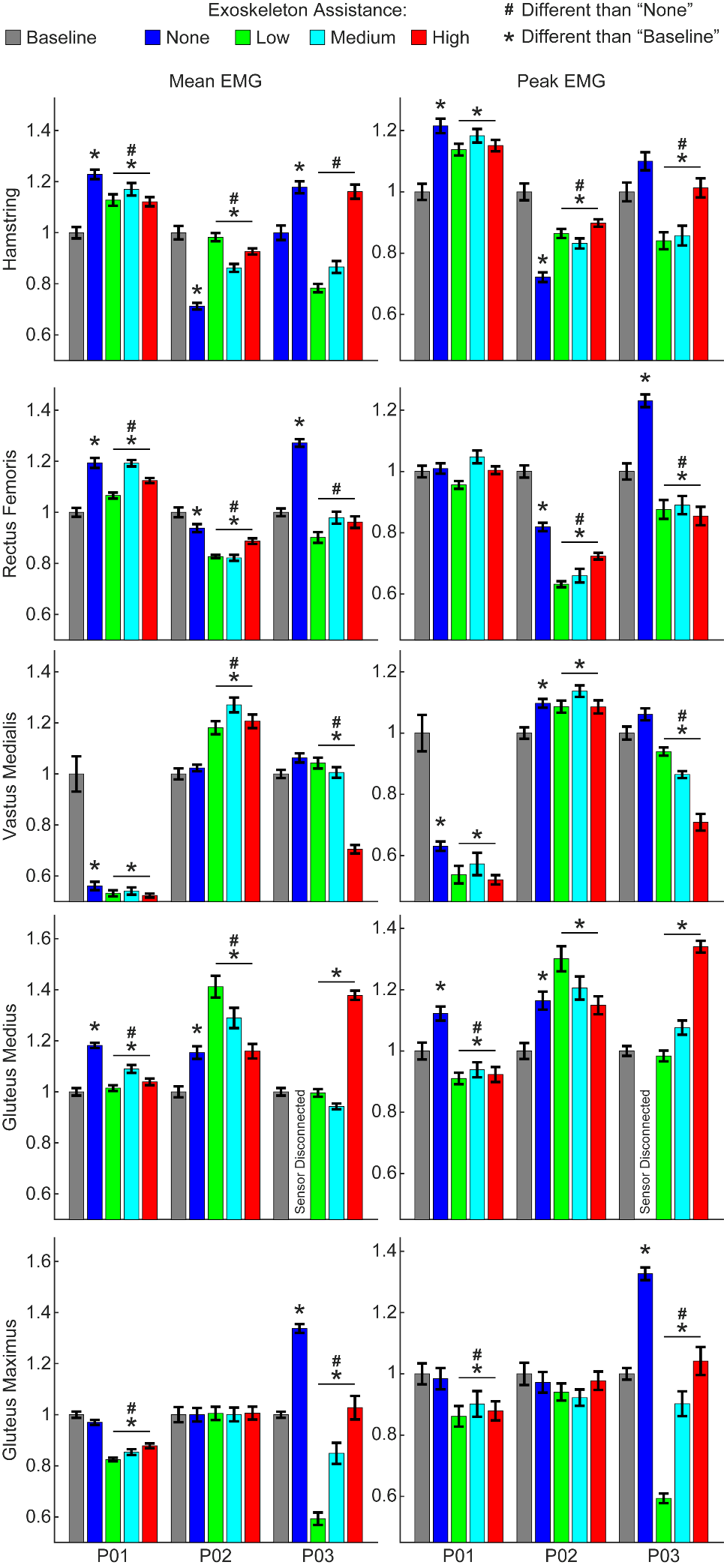


**Figure S1.** Changes in EMG activity due to exoskeleton assistance for the Hamstring, Rectus Femoris, Vastus Medialis, Gluteus Medius, and Gluteus Maximus. Changes in EMG activity for these muscles were generally nonlinear and unique to each participant. More consistent trends across participants were seen with the Soleus, Gastrocnemius and Erector Spinae (Fig. 2). Data show EMG activity averaged across each gait cycle for the right and left legs and normalized to the baseline condition.
